# A Phenomenological Approach to Study Mechanical Properties of Polymeric Porous Structures Processed Using Supercritical CO_2_

**DOI:** 10.3390/polym11030485

**Published:** 2019-03-13

**Authors:** Antonio Tabernero, Lucia Baldino, Stefano Cardea, Eva Martín del Valle, Ernesto Reverchon

**Affiliations:** 1 Department of Chemical Engineering, University of Salamanca, Plaza los Caídos s/n, 37008 Salamanca (SA), Spain; antaber@usal.es; 2 Department of Industrial Engineering, University of Salerno, Via Giovanni Paolo II, 132, 84084 Fisciano (SA), Italy; lbaldino@unisa.it (L.B.); ereverchon@unisa.it (E.R.); 3 Instituto de Investigación Biomédica de Salamanca, Hospital Virgen de la Vega, Paseo San Vicente, 58-182, 37007 Salamanca (SA), Spain

**Keywords:** supercritical CO_2_, hyperelasticity, strain energy functions, Ogden, Yeoh

## Abstract

This work proposes a modeling of the mechanical properties of porous polymers processed by scCO_2_, using a phenomenological approach. Tensile and compression tests of alginate/gelatin and cellulose acetate/graphene oxide were modeled using three hyperelastic equations, derived from strain energy functions. The proposed hyperelastic equations provide a fair good fit for mechanical behavior of the nanofibrous system alginate/gelatin (deviations lower than 10%); whereas, due to the presence of the solid in the polymer network, a four-parameter model must be used to fit the composite cellulose acetate/graphene oxide behavior. Larger deviations from the experimental data were observed for the system cellulose acetate/graphene oxide because of its microporous structure. A finite element method was, then, proposed to model both systems; it allowed a realistic description of observable displacements and effective stresses. The results indicate that materials processed using scCO_2_, when submitted to large stresses, do not obey Hooke´s law and must be considered as hyperelastic.

## 1. Introduction

Supercritical CO_2_ (scCO_2_) techniques have been used to process various polymers and to generate materials with appropriate properties to be used as membranes or scaffolds [1,2]. Among these, supercritical phase inversion and supercritical gel drying take advantage of the well-known properties of scCO_2_ (negligible surface tension, high diffusion coefficient, low viscosity, etc.) to produce materials with a 3D structure, proper porosity and, interconnected pores [3,4]. Therefore, these scCO_2_ techniques are adequate to manufacture polymers with better structural properties in a faster way than the conventional methodologies. The processed polymers can be a suitable platform for tissue engineering and regenerative medicine or for membranes applications, depending on the morphology and on the specific material properties [4,5]. Some reviews and articles have also been published covering scCO_2_ based processes, trying to highlight their theoretical basis and their advantages when compared to conventional techniques, such as freeze drying, phase separation or emulsion technologies [3,6,7]. For example, scCO_2_ has been previously used to dry a chitosan gel, removing, in addition, glutaraldehyde to avoid cito-toxicity problems, and obtaining useful material for tissue engineering applications [8]. Cellulose acetate membranes, loaded with different active agents, were obtained using a supercritical phase inversion technique [1].

With the aim of improving material properties, scCO_2_ based techniques can also be used to produce composites based on polymers containing nanoparticles in the network or an interpenetrated polymers network (IPN), in which both polymers are crosslinked to interlock both solid phases. Successful results were obtained in the case of cellulose acetate loaded with graphene oxide, for membrane applications [9] and when an IPN of alginate/gelatin was proposed, for tissue engineering applications [10].

Considering the properties of the generated structures, mechanical properties are relevant characteristics to be studied, to produce polymeric systems suitable for different applications. Compression and tensile mechanical tests have been performed on scCO_2_ processed materials, in order to assess their stress–strain response [2,5,11,12]. However, these results are usually expressed in terms of the Young modulus, which is generally calculated as the initial slope of the stress–strain deformation curve and takes into account that the material obeys the Hooke’s law, for small deformations, with a highly ideal and linear stress–strain behavior. This hypothesis can lead to erroneous interpretations of materials behavior, mainly at high stress values, if largely nonlinear responses are experimentally observed. A proper knowledge, based on the mechanical response, of the processed materials could be obtained using finite element modelling (FEM); however, it required a correct fitting of the stress–strain experimental data.

According to the literature, a nonlinear stress–strain deformation behavior has been observed for biological tissues (ligaments, tendons, blood vessels, etc.), gels and cross-linked polymers [13,14]. This evidence is related to viscoelastic effects or covalent bonds, that involve an initial strong stress to produce an initial deformation, commonly called toe region. Then, stress–strain follows a linear tendency until larger stresses are again required to produce a drastic increase of the deformation, previous to material failure. This kind of curve matches with the typical definition of hyperelastic materials [15]: Hyperelastic materials are elastic materials with a strong nonlinear stress–strain response. Although both elastic and hyperelastic solids involve a reversible deformation, elasticity deformation is always linear and it is the main difference between the nonlinear hyperelastic response. Hyperelasticy phenomenon is generally studied with a phenomenological approach that indicates that a material is hyperelastic if its stress–strain curve is successfully fitted by constitutive models called hyperelastic equations. These equations are characterized by several adjustable parameters and are derived from stress energy functions (W) that take into account energetic path relationships and correlate the strain energy of a material with the deformation gradient. A phenomenological approach, based on these strain energy functions, has been previously used to characterize biological tissues and elastomers [16,17].

Some different constitutive models can be used for modeling hyperelastic materials. A polynomial model, called generalized model, developed by Rivlin and Sanders [18,19], expresses the material behavior in terms of strain invariants of the Cauchy–Green deformation tensor. Later, Ogden introduced a strain energy function that depends on the principal stretches of the main axis [20]. At last, the Yeoh model only depends on the first strain invariant [21]. These constitutive models have been previously used to fit stress–strain data for an intervertebral disc polyurethane implant [22], for arterial layers with collagen fiber orientation [23] and for other biological tissues, highlighting their hyperelasticity behavior [16]. Specifically, for tensile tests, the Yeoh model was successfully used to fit experimental data for 3D printing [24]; whereas, it provided a worse prediction for some silicone gels [17]. On the other hand, for compressible loading, Ogden, Yeoh and the polynomial model were used to describe the mechanical properties of the human spinal cord [16]. In this case, the Yeoh model provided an appropriate fit. Ogden was successfully used to predict the mechanical properties, under a compression test, of polyurethane used as a model for the implant of a human intervertebral disc [22]. Finally, different hyperelastic models were defined to study the behavior of the system PDLL-nanohydroxyapatite [25].

Among the above mentioned models, the Ogden model is particularly relevant, since it is implemented in many commercial softwares for FEM application, such as ANSYS or FEBio. Therefore, if the stress–strain curve of the material is properly fitted using this model, the adjustable parameters can be subsequently introduced in the FEM software. The obtained material responses (effective stress, elongation, etc.), for any kind of stress, may be predicted using this simulation tool.

Stress–strain curves concerning porous structures processed by scCO_2_ have not been yet mathematically studied using a phenomenological approach or using a FEM model. To the best of our knowledge, only two papers studied the mechanical response of structures processed by scCO_2_; these works were produced to fit solid morphology using multi-scale modeling, without predicting stress–strain behavior. Although these results are able to explain solid characteristics with a correct Young modulus approximation, both solids were considered as elastics (linear stress–strain behavior), and stress–strain response at large stresses was not predicted [12,26,27].

Therefore, based on the previous discussion, and in order to acquire a better knowledge of the mechanical properties of porous polymers processed by scCO_2_, in this work, for the first time, the hypothesis that these materials can follow a hyperelastic behavior has been introduced. Solids incompressibility was taken into account to develop the hyperelastic equations to check that, in spite of the large porosity of some materials, this assumption can be acceptable to obtain a proper fit.

Specifically, a nanofibrous composite aerogel of an IPN alginate/gelatin (A/G) and a porous composite membrane of cellulose acetate/graphene oxide (CA/GO) will be considered, looking at tensile and compression tests, respectively. Their mechanical response will be fitted using hyperelastic constitutive models with three (Yeoh), four (Ogden) and five (generalized polynomial model) adjustable parameters. Then, a FEM model will be built, using the adjustable parameters obtained through the previous simulation.

## 2. Materials and Methods

### 2.1. Preparation of the Materials and Characterizations

The materials used, the methodology to produce the different systems and the characterization techniques were already discussed elsewhere [9,10]. Basically, the supercritical-assisted phase inversion process was used to obtain nanocomposite membranes of cellulose acetate loaded with different concentrations (0%, 3% and 9% *w*/*w* with respect to CA) of graphene oxide (CA/GO) [9].

The experiments started with the preparation of the solutions of CA (25% *w*/*w*) in *N*-methylpyrrolidine. The GO was added to the solution, and the formed suspension was introduced in a steel cap where the phase separation took place with scCO_2_ at 200 bars and 40 °C for 4 h (CO_2_ flow rate of 1.5 kg/h). Those conditions were selected to obtain homogeneous membranes with regular pore morphology. The addition of GO increased the pore size (from 9 to 14 nm) and the porosity (from 85 to 90%) [9].

Alginate/gelatin (A/G) aerogels were obtained after performing supercritical drying of an interpenetrated polymer network of alginate/gelatin hydrogels, that were produced from solutions at 2% *w*/*w* or 5% *w*/*w* of the two bio-polymers [10]. The solutions were mixed in different A/G ratios: 20/80, 50/50, 80/20 and were poured into a mold that was put in contact with a solution of CaCl_2_ to crosslink the alginate and then with a solution of glutaraldehyde to crosslink the gelatin. The obtained hydrogel was then introduced in a vessel, where scCO_2_ drying was performed from 5–8 h (flow rate about 1 kg/h) with a pressure of 200 bars and 35 °C to produce the aerogels. These materials were characterized by a nanofibrous structure and the porosity increases, from 88 to 93%, with the amount of gelatin [10].

### 2.2. Mechanical Tests

Mechanical properties of the structures were studied using an INSTRON 4301 (Instron Int. Ltd., High Wycombe, UK). The results concerning the compression test for the system CA/GO were already published [12]; whereas, uniaxial tensile tests were specifically performed for the system A/G to enlarge the number of mechanical tests data to perform for the fitting procedure. In this last case, samples with a length of 3.5 cm and 1.5 cm of thickness were previously immersed in water for 20 h. After that, the tensile response was studied using a 100 N load cell, at 1.5 mm/min and at room temperature.

### 2.3. Models Development

**Figure 1:**
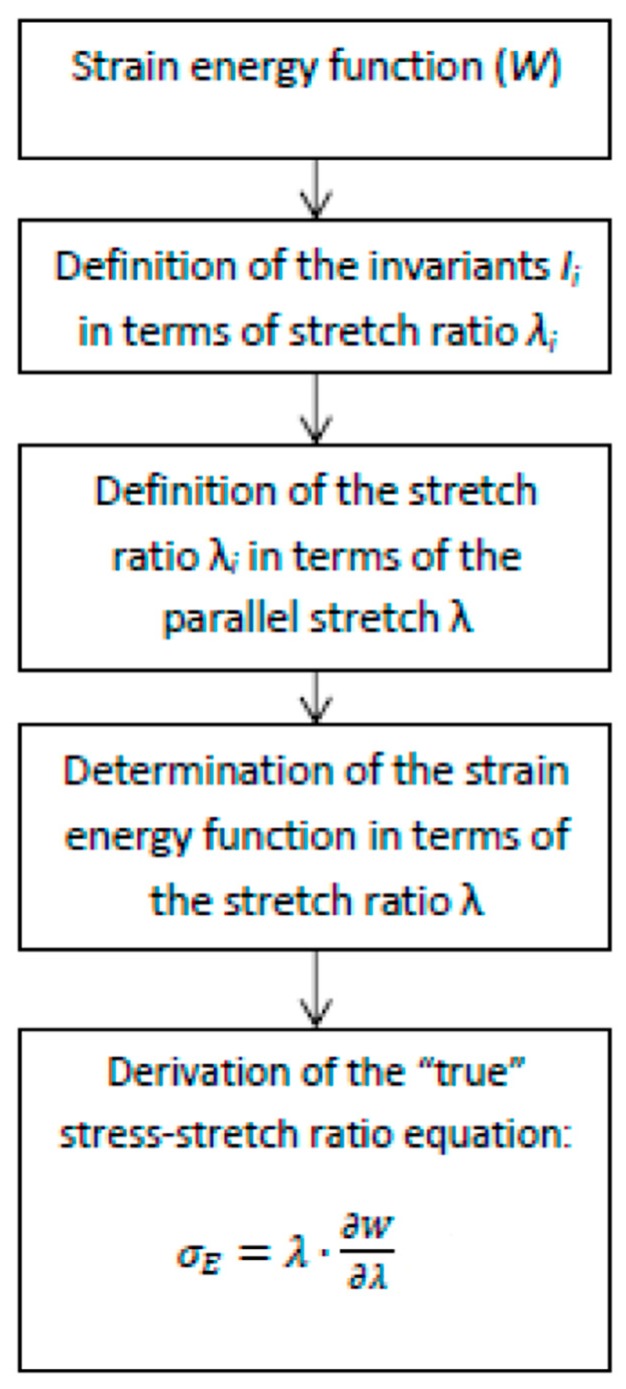
Steps for deriving stress–stretch equation.

Three different strain energy functions were used for modeling materials stress–strain responses. These models establish a relationship stress–stretch ratio based on an energy function that can be expanded in a mathematical series. The steps to derive a stress–stretch relationship from stress energy functions are described in Figure 1. More detailed information to derive these kind of equations is given in [22,28]. Strain energy functions are generally expressed in terms of the invariants of the Cauchy tensor or in terms of the main stretch ratio (x, y and z have each one a different stretch ratio). These stretch ratios have to be expressed in terms of a main (or parallel) stretch ratio. The main stretch ratio for a tensile test will be in the x axis (positive); whereas, for a compression test, it will be in the y axis (negative). Once the strain energy function is expressed in terms of the main stretch ratio, the stress–stretch equation will be derived:

Prior to the development of the models, it is important to explain the definition of the stretch ratio (*λ_i_*) and the different relationships between invariants and stretch ratios. Stretch ratio is defined as the ratio of the deformed length *l_i_* to the undeformed length *L_i_* in the principal axes (Equation (1)).

(1) λi=liLi

The strain invariants can be expressed in terms of the stretch ratios *λ*_1_, *λ*_2_, *λ*_3_ [29]. Despite the fact that the CA/GO material is porous, as a first approximation, both polymeric mixtures were considered as incompressible to calculate the invariant *I*_3_. Finally, for uniaxial mechanical tests, it is possible to define the different stretch ratios (*λ*_1_, *λ*_2_, *λ*_3_) in terms of the parallel stretch *λ*. This methodology to derive the hyperelastic equations from energy functions is explained with detail in the supplementary material (S1). This mathematical procedure has been used before by some authors [22,25].

#### 2.3.1. Generalized Polynomial Model

This model is dependent on the invariant tensors *I*_1_ and *I*_2_ for the Cauchy–Green deformation tensor (Equation (2)):

(2) $$W=\sum_{i=1,j=1}^{n}C_{ij}\cdot {\left(I_{1}-3\right)}^{i}\cdot {\left(I_{2}-3\right)}^{j},\;\mathrm{where}\;C_{00}=0$$

This equation was developed for five parameters. After substituting the invariants in terms of the parallel stretch ratios, the strain energy function can be derived to obtain the relationship stress–stretch ratio using the equation in Figure 1. The final equation (named polynomial model) is (Equation (3)):

(3) $$\sigma_{E}\left(\lambda \right)=2\cdot \left(\lambda -\lambda^{-2}\right)\cdot \left[C_{10}+C_{01}\cdot \lambda^{-1}+2C_{20}\left(\lambda^{2}+2\lambda^{-1}-3\right)+2C_{02}\left(2\lambda +\lambda^{-2}-3\right)\;+3C_{11}\left(\lambda -1-\lambda^{-1}+\lambda^{-2}\right)\right]$$

The final equation is a polynomial model with five adjustable parameters *C*_10_, *C*_01_, *C*_20_, *C*_02_, *C*_11_, in which the stress may be calculated depending on the stretch ratio.

#### 2.3.2. Ogden Model

Ogden model (Equation (4)) establishes a relationship between the stress and the stretch ratio and, therefore, it is not required to substitute the invariants in terms of the different stretch ratios:

(4) $$W=\sum_{p=1}^{n}\frac{\mu_{P}}{\alpha_{P}}\cdot \left(\lambda_{1}^{\alpha_{P}}+\lambda_{2}^{\alpha_{P}}+\lambda_{3}^{\alpha_{P}}-3\right)$$

In order to obtain a model with four adjustable parameters (*α*_1_, *α*_2_, *μ*_1_ and *μ*_2_), the model was developed by taking into account *n* = 2. Finally, after substituting the main stretch ratios by the parallel stretch ratios and deriving the resulting equation using in Figure 1, it is possible to obtain the final Ogden model stress–stretch ratio (Equation (5)):

(5) $$\sigma_{E}\left(\lambda \right)=\frac{1}{\lambda^{2}}\cdot \left(\mu_{1}\cdot \lambda^{\alpha_{1}}+\mu_{2}\cdot \lambda^{\alpha_{2}}-\mu_{1}\cdot \lambda^{\left(-\frac{1}{2}\right)\alpha_{1}}-\mu_{2}\cdot \lambda^{\left(-1/2\right)\alpha_{2}}\right)$$

#### 2.3.3. Yeoh Model

Yeoh introduced a model that only depends on the first strain invariant (Equation (6)). This model is also based on a series expansion and it is generally developed for three adjustable parameters:

(6) $$W=\sum_{i=1}^{n}C_{i}\cdot {\left(I_{1}-3\right)}^{i}$$

After substituting the invariants in terms of the main parallel stretch and using the equation in Figure 1, the final equation stress–stretch (Equation (7)) for *n* = 3 is derived and Yeoh model is obtained with three adjustable parameters *C*_1_, *C*_2_ and *C*_3_.

(7) $$\sigma_{E}\left(\lambda \right)=2\cdot C_{1}\cdot \left(\lambda -\lambda^{-2}\right)+4\cdot C_{2}\cdot \left(\lambda -\lambda^{-2}\right)\cdot \left(\lambda^{2}+2\lambda^{-1}-3\right)+6\cdot C_{3}\cdot \left(\lambda -\lambda^{-2}\right)\cdot {\;\left(\lambda^{2}+2\lambda^{-1}-3\right)}^{2}$$

#### 2.3.4. Transformation Engineering Stress-True Stress

Equations (3), (5) and (7) show true stress (*σ_E_*)–stretch ratio relationship. These equations have a different number of adjustable parameters that must be obtained by fitting experimental data–theoretical data. However, tensile and mechanical tests provide experimental data in terms of displacement and engineering stress, that is calculated as the load divided by the original cross-sectional area. These mechanical experiments do not give information concerning true stress (*σ_E_*) since phenomena, such as necking in tensile test (area reduction) and the area increase in compression tests, are not taken into account. The true stress and engineering stress relationship is the following (Equation (8)) [30,31]:

(8) $$\sigma_{E}=\sigma \cdot \left(1+\lambda \right)$$

Therefore, some papers show their results in terms of true stress and the term (1 + *λ*) [22,23]. This term (1 + *λ*) is always larger than one for a tensile test; whereas, it is lower than 1 for a compression test. In addition, true stress is negative for a compression test (the displacement is on the negative y axis); whereas, true stress for tensile tests is positive (displacement on the positive x axis).

### 2.4. FEM Modeling

FEM modeling was performed using FEBio [32], a finite element modeling software that was developed to study nonlinear finite element problems in biomechanics, where biological tissues provide strong nonlinear stress–strain responses. For this reason, some hyperelastic models, such as the Ogden model, are implemented in this software.

#### 2.4.1. FEM Modeling for Tensile Test

To perform a tensile test, a sample of a material is drawn. It is subsequently meshed with hexaedral elements with *n*x = 20, *n*y = 10 and *n*z = 1. Then, in order to study the effective stress due to a tensile test, a displacement of 1 unit in the positive x axis was prescribed; whereas, the displacements on the opposite side are fixed to 0 (to create an effect similar of pulling from a fixed bar). Finally, Ogden’s parameters are included in the material definition and the simulation is performed.

#### 2.4.2. FEM Modeling for Compression Test

A cube of known dimensions is drawn and meshed subsequently, in this case with butterfly elements with *n*x = 10, *n*y = 10, *n*z = 10, *n*seq = 10 and a ratio of 0.5. Then, a 1/4 of the meshed geometry is extracted to reduce the number of simulations because the results will be geometrically similar. After fixing the displacements in the respective axis, a wall contact is created on the top of the surface to simulate the movement of the cross-area in the negative y axis. As with the tensile tests, Ogden´s parameters are given for the material definition and the simulation is run. The results will highlight the effective stress tensor and the displacement on the y axis.

## 3. Results and Discussion

### 3.1. Morphological Results

**Figure 2:**
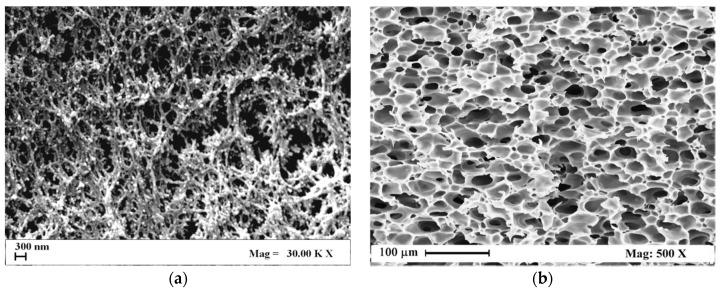
FE-SEM pictures of samples internal section: (**a**) A/G 50/50% *v*/*v* aerogel and (**b**) CA/GO 9% *w*/*w* membrane.

This part of the work is related in part to data proposed in previous works [9,10] and here further developed, and in part to experimental results, specifically performed. For this purpose, aerogels of alginate with gelatin (A/G) and membranes of cellulose acetate with graphene oxide (CA/GO), have been characterized from a mechanical point of view. In particular, two different procedures were adopted: the first one was applied to tensile tests for the A/G aerogels; the second one was a compression test for CA/GO membranes. In both cases, a scCO_2_ assisted process was used to obtain solvent free polymer samples that maintained their porous structure organized at nano- and at micro-scale thanks to the peculiarities of the supercritical mixture (CO_2_ + organic solvent); i.e., near zero surface tension, when opportune process conditions were adopted. Figure 2 reports two SEM pictures related to the morphology of an A/G aerogel (Figure 2a) and of a CA/GO membrane (Figure 2b). It is clearly visible that different morphologies were obtained as previously described [9,10]: it was nanofibrous in the first case [10]; microporous in the second one [9]. These two different polymer systems allow the application of hyperelastic models from nanoscale to microscale, when tensile or compression tests are available or specifically produced.

### 3.2. Modeling Experimental Data Using Hyperelastic Equations

#### 3.2.1. Tensile Tests

**Figure 3:**
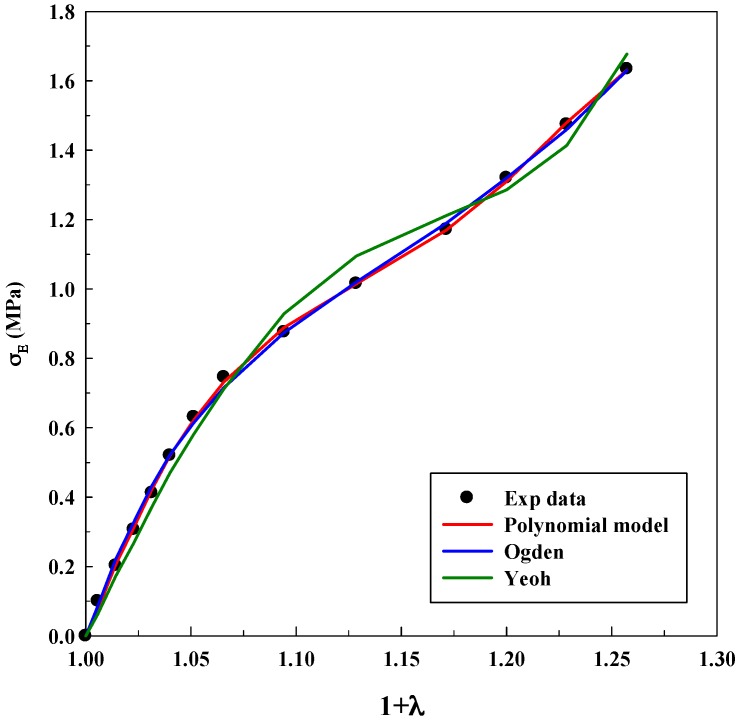
Models fitting of stress–elongation for the system A/G 20/80% *v*/*v* at 2% *w*/*w*.

**Figure 4:**
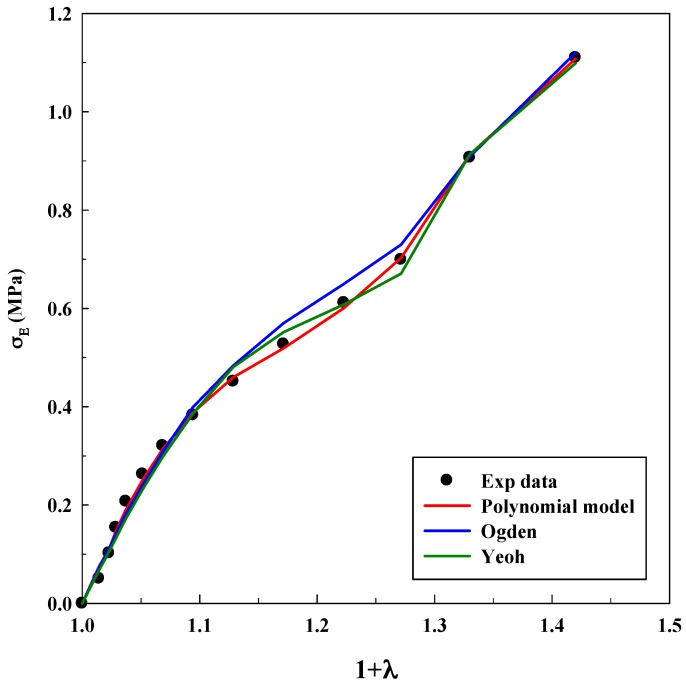
Models fitting of stress–elongation for the system A/G 50/50% *v*/*v* at 2% *w*/*w*.

**Figure 5:**
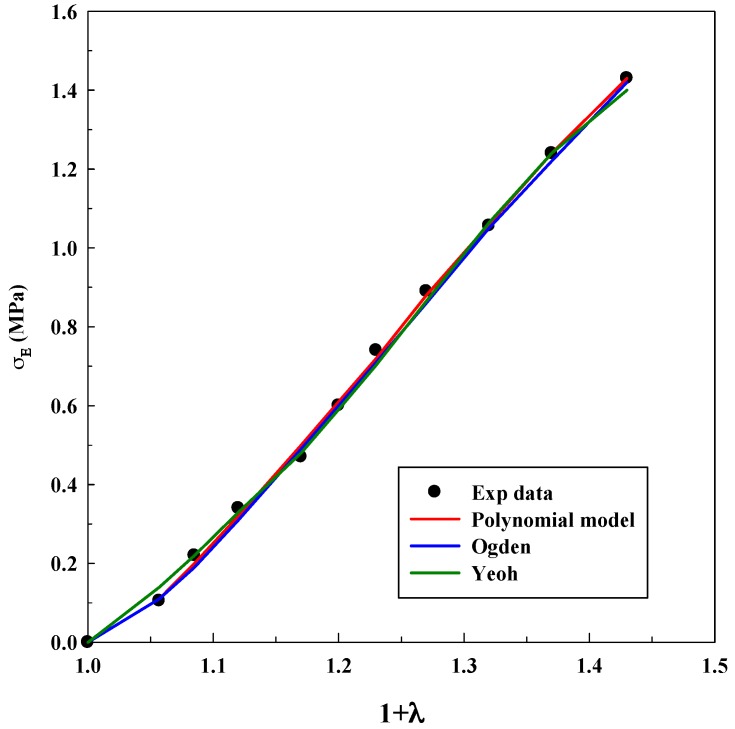
Models fitting of stress–elongation for the system A/G 50/50% *v*/*v* at 5% *w*/*w*.

**Figure 6:**
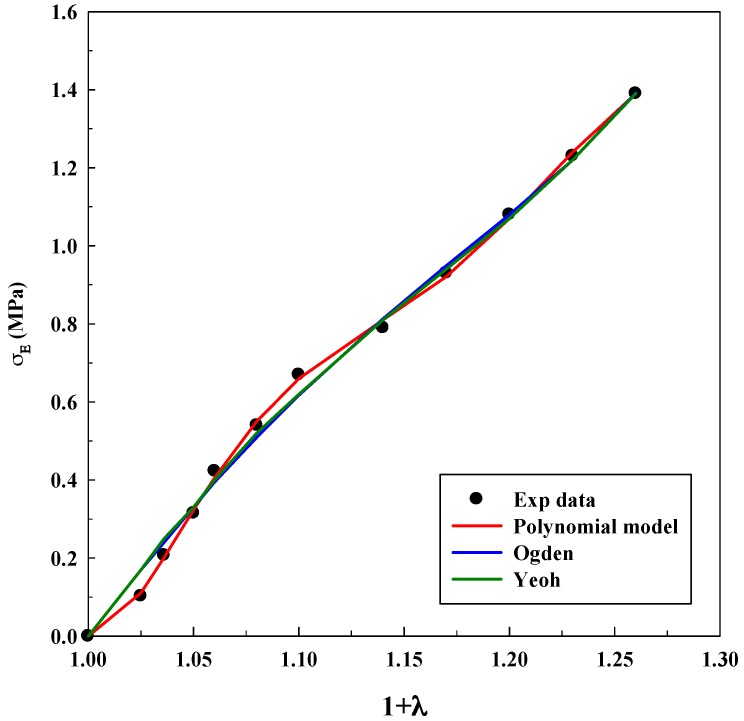
Models fitting of stress–elongation for the system A/G 80/20% *v*/*v* at 2% *w*/*w*.

**Figure 7:**
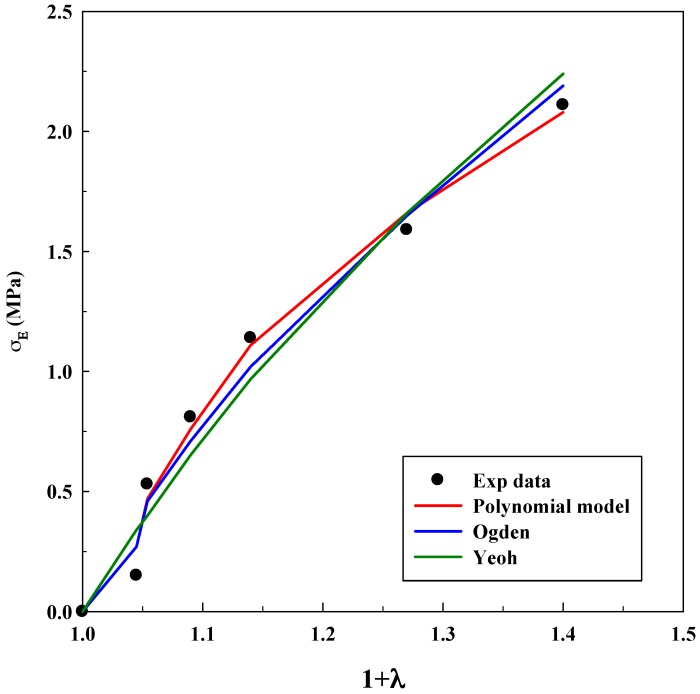
Models fitting of stress–elongation for the system A/G 80/20% *v*/*v* at 5% *w*/*w*.

As it was explained before, Equations (3), (5) and (7) were used for modeling experimental data to observe true stress–elongation (1 + *λ*). The average absolute relative deviation was calculated to determine the fit accuracy (see supplementary material (S2)). In this case, it is relevant to realize that the experimental data related to true stress at 0 MPa was not taken into account to avoid mathematical indeterminations. Table 1, Table 2 and Table 3 show the adjustable parameters for the three equations and the fitting deviation. Figure 3, Figure 4, Figure 5, Figure 6 and Figure 7 contain the results obtained.

Figure 3, Figure 4, Figure 5, Figure 6 and Figure 7 allow to observe that stress–elongation curves for a tensile test of this material are characterized by different behaviors. Curves are formed by a region in which an initial stress is required to produce an elongation. That region is called toe region and is attributed to the forced alignment of the different polymeric chains and/or covalent bonds. Then, there is a linear region followed by another curve previous material failure. Depending on the processed material, the toe region can encompass different values of stress, generally around 0.025 units of elongation in the studied materials, with different behaviors.

In the case of the system alginate/gelatin, an increase of the gelatin percentage (i.e., from 20 to 80 *v*/*v*) produces an additional and larger resistance for the initial elongation. Indeed, as it can be observed in Figure 3, Figure 4 and Figure 6, related to different A/G ratio systems and to 2% *w*/*w* of polymers, a stress of 0.3 MPa is required to produce an initial elongation of 1.025 for the system A/G 20/80% *v*/*v*; whereas, 0.1 MPa are needed for producing the same elongation (1.025) for the system A/G 50/50% and around 0.08 MPa for A/G 80/20%. This fact involves a modification of the stress–elongation curve and, as a consequence, a different mechanical behavior. As it can be expected, an increase in the gelatin percentage increases the required stress for an initial elongation; indeed, gelatin is a product of collagen degradation, that confers mechanical resistance to the polymeric mixture. The same phenomenon happens at 5% *w*/*w* because a stress of 0.12 MPa is required to produce an elongation of 1.025 (A/G 50/50%, Figure 5) whereas 0.1 MPa is needed for 1.025 when the composition is A/G 80/20% (Figure 7).

Moreover, an increase of the total concentration (from 2% *w*/*w* to 5% *w*/*w*) of the solids (for the same A/G composition), increases the required stress for straining the IPN previous material failure.

The Yeoh, Ogden and polynomial models properly fit the experimental data for all the investigated conditions for the system A/G, with deviations, generally, lower than 10%. However, it is important to specify that there is not a significant decrease in the deviation when the number of parameters of the models increases. An acceptable deviation is obtained using the Yeoh model (only three parameters) and, as a consequence, it can be properly used for estimating stress–strain behavior of this system. This is an advantage, since a reduced number of parameters avoids possible problems concerning convergency or number of iterations of the numerical method. The Ogden model with four parameters and polynomial model with five parameters provide similar deviations (although slightly lower than Yeoh). Numerical value of the model parameters might be an important issue to study future material changes, since they will be modified by polymer lifetime, as it has been observed in real (biological) systems for the osteoarthritic human articular cartilage [33]. However, in this study, it was not observed a precise tendency in the numerical value of the parameters with an increase of the gelatin in the polymeric mixture.

As it was mentioned in the Introduction, the Yeoh model was also able to model successfully the tensile test for materials with hyperelastic behavior, as materials for 3D printing and for biological systems [16,24].

The results of this study confirm that IPNs processed using scCO_2_ based technique behave as hyperelastic solids. Considering them as elastic materials can lead to large deviations in stress–strain response modeling at large stress.

**Figure 8:**
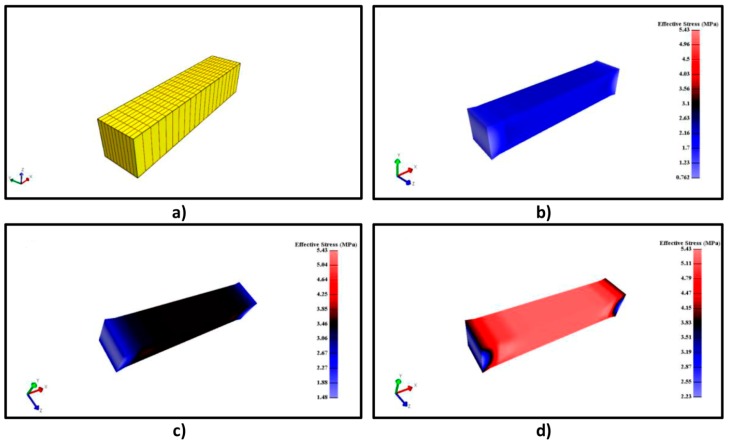
(**a**) Meshed geometry; (**b**) FEM simulation results for A/G 50/50% *v*/*v* at 2% *w*/*w*; (**c**) FEM simulation results for A/G 50/50% *v*/*v* at 5% *w*/*w*; (**d**) FEM simulation results for A/G 80/20% *v*/*v* at 5% *w*/*w*.

Ogden simulations are relevant since this equation is implemented in some FEM software. Deviations lower than 10% were obtained using this equation and, therefore, it is used for material definition in the following FEM simulation process. FEM modeling results for the systems A/G 50/50% *v*/*v* (2% *w*/*w*), A/G 50/50% *v*/*v* (5% *w*/*w*) and A/G 80/20% *v*/*v* (5% *w*/*w*) can be observed in Figure 8. Figure 8a shows the material organization after the meshing process; its geometry was meshed in 462 nodes with 200 elements. Figure 8 illustrates the results of the FEM analysis on the described material. As it was explained in Section 2.4.1, an elongation of 1 unit was fixed in the preprocessing step. This elongation provides, as a consequence, an effective stress on the material on the x axis. The numerical value of this stress is obtained from the simulation and it is illustrated in Figure 8b–d, that report result for the above mentioned elongation of 1 unit for three different materials. The effective stress obtained from the simulation is similar to the observed experimental stress. Around 0.8 MPa are needed for 0.30 units of elongation for Figure 8b (see Figure 4) and 1 MPa and 1.70 MPa are required for the same elongation in Figure 8c (see Figure 5) and Figure 8d (see Figure 7), respectively. Therefore, FEM is useful to predict the material behavior depending on the composition of the different systems. Figure 8 shows in addition that the highest stress value for every material (2.2 MPa, 3.63 MPa and 5.43 MPa) is found in the middle of the material, where the necking phenomenon takes place. These results highlight that the Ogden model and previous fitting of stress–elongation data can be used to perform a proper FEM modeling. The same modeling can be useful to extrapolate the different mechanical responses such as effective stress on any axis, total effective stress, displacements or relative volume. Therefore, it would be possible to acquire a better knowledge of the material.

#### 3.2.2. Compression Tests

**Figure 9:**
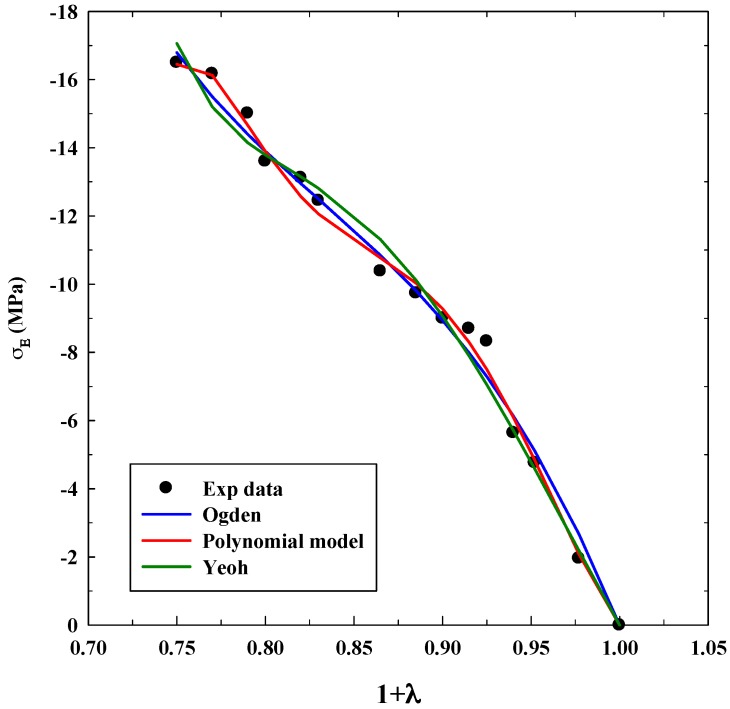
Fitting of compression tests for the system CA without GO using different models.

**Figure 10:**
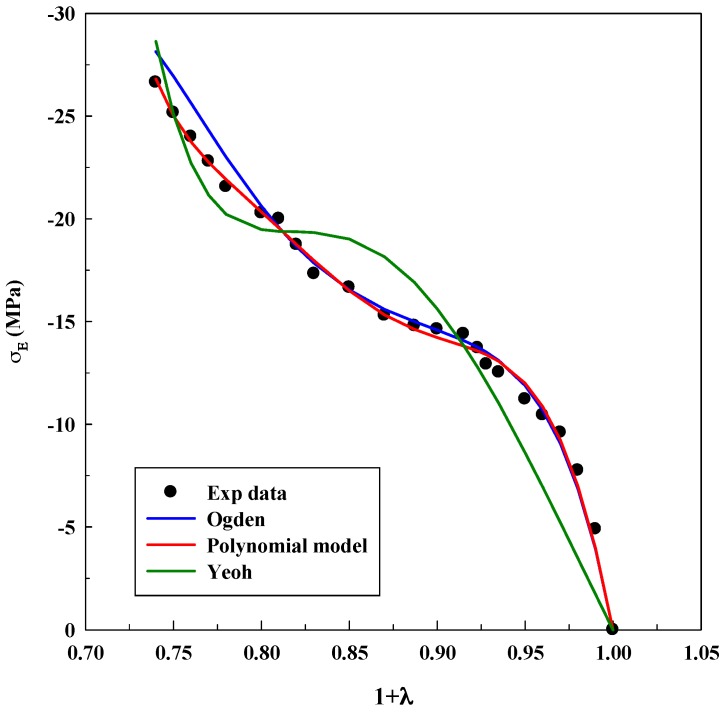
Fitting of compression tests for the system CA loaded with 3% *w*/*w* GO using different models.

**Figure 11:**
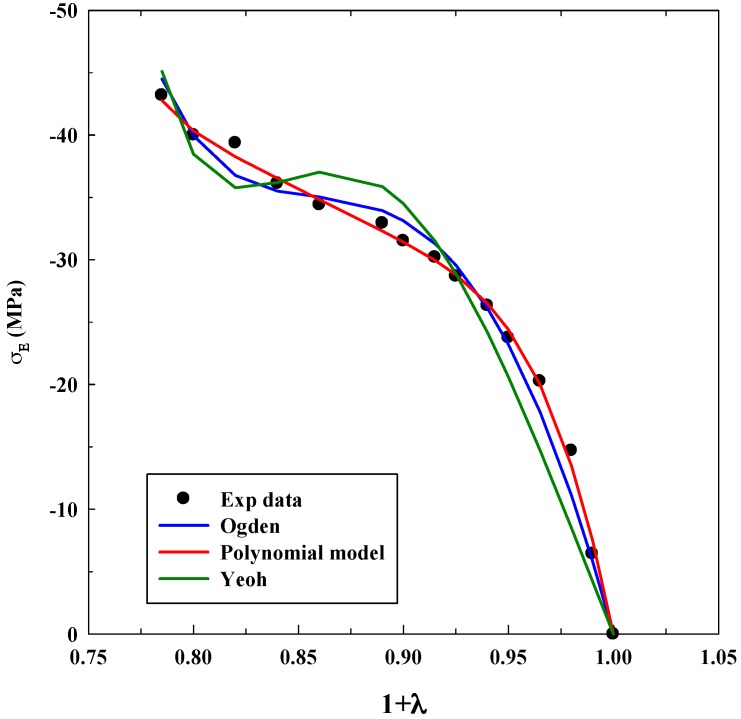
Fitting of compression tests for the system CA loaded with 9% *w*/*w* GO using different models.

As it was done for the tensile test, Table 4, Table 5 and Table 6 show the parameters for the system CA/GO after the fitting process. Figure 9, Figure 10 and Figure 11 compare experimental and numerical data.

Experimental results show cellulose acetate (CA) compressive resistance and its displacement for a given load. Graphene oxide (GO) increases solids microporosity as well as their resistance to a great extent. Toe area increases from −8 MPa (without GO) until −30 MPa (with 9% *w*/*w* GO). Furthermore, the linear region decreases when the concentration of GO increases. Microporosity produces a more compressible solid, which is the reason why the models will produce larger deviations (incompressibility is assumed for models development). For this reason, the Yeoh model can only successfully fit the system without GO (as can be seen in Figure 9); whereas, it fails when GO is included in the material (Figure 10 and Figure 11): this equation cannot describe systems with this microporosity value, which increases the material compressibility.

The Ogden model and polynomial model fit the experimental data in a similar manner. This result indicates that at least four parameters are needed to fit properly the mechanical response of this material and to mathematically compensate the incompressibility hypothesis. Deviations are slightly higher for CA/GO than for the system A/G because the loading of solid particles increases the compressibility value; toe and linear areas undergo abrupt changes when compared to the system without GO. Again, a precise tendency is not observed in the values of the adjustable parameters. Only an increase in the Yeoh model parameters when GO concentration increases was observed, although this increment is not dependent on the concentration of GO.

The results for compression tests highlight that, as happened with the previous IPN, the polymer and the polymer loaded with nanoparticles, processed by scCO_2_, behave as a hyperelastic material and that an elastic approach is not suitable for studying its mechanical response at high stresses. However, as it was expected, deviations increase when porous solids are studied.

**Figure 12:**
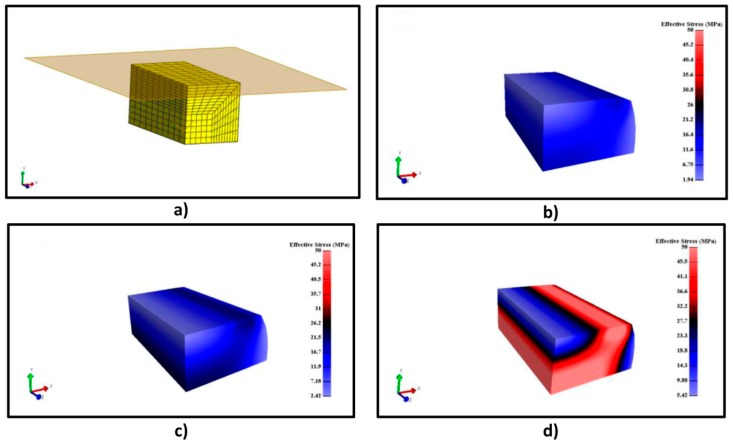
(**a**) Meshed geometry; (**b**) FEM simulation results for CA without GO; (**c**) FEM simulation results for CA with 3% GO; (**d**) FEM simulation results for CA with 9% GO.

FEM simulation of the compression test was performed using the Ogden model parameters. Figure 12a shows the meshed geometry, with the wall contact to create the movement on the y axis, using 1606 nodes and 1250 elements. Figure 12b–d illustrate FEM modeling results of the system of CA without GO and CA with 3% and 9% GO (referred to 1/4 of the whole sample and the results will be similar in a geometrical way). The wall contact movement compresses the materials to produce a stress and a displacement on the y axis. Figure 12b–d show the effective stress necessary for a displacement of −0.25 units for each system. It is possible to observe that, the effective stress distribution is similar for every material, although it increases when the GO concentration increases, as it was showed by the experimental results. It is also important to specify that the effective stress has a positive value; whereas, it was negative in Figure 10, Figure 11 and Figure 12. The explanation for this fact is that the effective stress is calculated as the square root of a combination of the product of different stresses, and it is always positive [32]. These results highlight that this model can be used for predicting the mechanical behavior of this material related to the produced stress.

## 4. Conclusions

In this work a phenomenological approach has been proposed to study mechanical properties of different polymer-based materials processed by scCO_2_. Models used provided an appropriate fit for the tensile test with deviations always smaller than 10%, showing that the use of the Yeoh model is enough to represent this kind of experimental data (nanofibrous structure). In the case of CA polymer loaded with GO particles, the Yeoh model fails to predict compressible solids (microporous structure), and four-parameter models are at least needed to obtain a correct fit.

As a final result, FEM modeling was used to calculate the effective stress tensor in a realistic way.

In conclusion, materials processed using scCO_2_ behave as hyperelastic materials and linear considerations about stress–deformation curves should be avoided, to get a proper knowledge in applications where large stresses are required.

## Figures and Tables

**Table 1:** Parameters and deviations for the Yeoh model.

Material	*C*_1_ (MPa)	*C*_2_ (MPa)	*C*_3_ (MPa)	AARD (%)
A/G (20/80% *v*/*v*) 2% *w*/*w*	2.09	−6.62	17.23	8.21
A/G (50/50% *v*/*v*) 2% *w*/*w*	0.81	−1.51	2.49	7.51
A/G (50/50% *v*/*v*) 5% *w*/*w*	0.44	0.79	-0.67	5.71
A/G (80/20% *v*/*v*) 2% *w*/*w*	1.19	−1.14	3.42	9.22
A/G (80/20% *v*/*v*) 5% *w*/*w*	1.32	−0.13	0.092	9.15

**Table 2:** Parameters and deviations for the Ogden model.

Material	*μ*_1_ (MPa)	*μ*_2_ (MPa)	*α*_1_	*α*_2_	AARD (%)
A/G (20/80% *v*/*v*) 2% *w*/*w*	−3.57	−72,658.00	−8.81	0.00027	3.07
A/G (50/50% *v*/*v*) 2% *w*/*w*	184.74	6.58	0.016	0.018	8.93
A/G (50/50% *v*/*v*) 5% *w*/*w*	5571.26	2101.80	0.050	−0.14	4.04
A/G (80/20% *v*/*v*) 2% *w*/*w*	1.95	−0.000021	2.50	−78.51	9.45
A/G (80/20% *v*/*v*) 5% *w*/*w*	451,719	0.190	0.000011	0.190	8.43

**Table 3:** Parameters and deviations for the polynomial model.

Material	*C*_10_ (MPa)	*C*_01_ (MPa)	*C*_20_ (MPa)	*C*_02_ (MPa)	*C*_11_ (MPa)	AARD (%)
A/G (20/80% *v*/*v*) 2% *w*/*w*	−2.02	4.60	−706.81	4426.25	−3740.65	2.40
A/G (50/50% *v*/*v*) 2% *w*/*w*	1.89	−0.98	−152.39	1012.33	−868.48	6.56
A/G (50/50% *v*/*v*) 5% *w*/*w*	4.05	−3.86	−11.98	87.91	−77.87	3.95
A/G (80/20% *v*/*v*) 2% *w*/*w*	44.88	−44.94	−1470.64	9611.43	−8226.17	2.10
A/G (80/20% *v*/*v*) 5% *w*/*w*	6.31	−4.93	−19.50	187.97	−175.38	6.67

**Table 4:** Parameters and deviations for the Yeoh model.

Material	*C*_1_ (MPa)	*C*_2_ (MPa)	*C*_3_ (MPa)	AARD (%)
CA	15.68	−35.62	56.89	4.96
CA (3% *w*/*w* GO)	28.65	−89.51	156.09	14.75
CA (9% *w*/*w* GO)	70.21	−327.78	795.86	10.84

**Table 5:** Parameters and deviations for the Ogden model.

Material	*μ*_1_ (MPa)	*μ*_2_ (MPa)	*α*_1_	*α*_2_	AARD (%)
CA	18.92	21.66	9.18	−4.17	6.06
CA (3% *w*/*w* GO)	21.42	20.35	25.86	−13.06	3.60
CA (9% *w*/*w* GO)	44.60	23.91	41.78	−12.10	2.53

**Table 6:** Parameters and deviations for the polynomial model.

Material	*C*_10_ (MPa)	*C*_01_ (MPa)	*C*_20_ (MPa)	*C*_02_ (MPa)	*C*_11_ (MPa)	AARD (%)
CA	−192.30	203.68	−12,033.70	46,593.40	35,041.82	3.53
CA (3% *w*/*w* GO)	1086.30	−1011.29	16,952.70	−61,260.80	45,493.80	3.01
CA (9% *w*/*w* GO)	1873.10	−1732.28	35,351.70	−132,802.01	99,498.93	2.52

## Supplementary Materials

Supplementary Materials are available online at https://www.mdpi.com/2073-4360/11/3/485/s1.
