# Supplementary material for: A Phenomenological Approach to Study Mechanical Properties of Polymeric Porous Structures Processed Using Supercritical CO2
Source: Polymers (Basel). 2019 Mar 13;11(3):485. doi: 10.3390/polym11030485 (PMC6473646; doi:10.3390/polym11030485)
Supplement: Supplementary file 1 [file polymers-11-00485-s001.pdf]

# Supplementary material

## S.1. Equations for models development

The strain invariants can be expressed in terms of the stretch ratios $\lambda_1$, $\lambda_2$, $\lambda_3$ (eq. 1, 2 and 3) [1]:

$$I_1 = \lambda_1^2 + \lambda_2^2 + \lambda_3^2 \quad (1)$$

$$I_2 = \lambda_1^2 \cdot \lambda_2^2 + \lambda_2^2 \cdot \lambda_3^2 + \lambda_3^2 \cdot \lambda_1^2 \quad (2)$$

$$I_3 = \lambda_1^2 \cdot \lambda_2^2 \cdot \lambda_3^2 \quad (3)$$

In our case, despite the fact that the CA/GO material is porous, as a first approximation, both polymeric mixtures were considered as incompressible; therefore, $I_3$ can be calculated as [1]:

$$I_3 = \lambda_1^2 \cdot \lambda_2^2 \cdot \lambda_3^2 = 1 \quad (4)$$

For uniaxial mechanical tests, it is possible to define the different stretch ratios ($\lambda_1$, $\lambda_2$, $\lambda_3$) in terms of the parallel stretch $\lambda$ [1].

For a tensile test (eq. 5, 6 and 7) the main stretch is $\lambda_1$ (x axis):

$$\lambda_1 = \lambda \quad (5)$$

$$\lambda_2 = \lambda_3 \quad (6)$$

$$\lambda_2 = \lambda_3 = \lambda^{1/2} \quad (7)$$

Whereas, for a compression tests (eq. 8, 9 and 10) the main stretch is $\lambda_2$ (y axis):

$$\lambda_2 = \lambda \quad (8)$$

$$\lambda_1 = \lambda_3 \quad (9)$$

$$\lambda_1 = \lambda_3 = \lambda^{1/2} \quad (10)$$

## S.2. Equation for determining the average absolute relative deviation

$$AARD\left(\%\right)=\frac{100}{n}\cdot\sum\frac{\sigma_E^{calculated}-\sigma_E^{\exp erimental}}{\sigma_E^{\exp erimental}} \quad (11)$$


1. Treloar, L.R.G. The elasticity and related properties of rubbers. *Rep. Prog. Phys.* **1973**, *36*, 755–826.
